# RETRACTED: Quazi, S.; Malik, J.A. A Systematic Review of Personalized Health Applications through Human–Computer Interactions (HCI) on Cardiovascular Health Optimization. *J. Cardiovasc. Dev. Dis.* 2022, *9*, 273

**DOI:** 10.3390/jcdd12110446

**Published:** 2025-11-17

**Authors:** Sameer Quazi, Javid Ahmad Malik

**Affiliations:** 1 GenLab Biosolutions Private Limited, Bangalore 560043, Karnataka, India; 2 Department of Biomedical Sciences, School of Life Sciences, Anglia Ruskin University, Cambridge CB1 1PT, UK; 3 Clinical Bioinformatics, School of Health Sciences, The University of Manchester, Manchester M13 9P, UK; 4 Department of Zoology, Guru Ghasidas University, Bilaspur 495009, Chhattisgarh, India

The Journal of Cardiovascular Development and Disease retracts the article “A Systematic Review of Personalized Health Applications through Human–Computer Interactions (HCI) on Cardiovascular Health Optimization” [1], cited above.

Following publication, concerns were brought to the attention of the Editorial Office regarding the authorship list and origins of this study [1].

Adhering to our standard procedure, the Editorial Office and Editorial Board conducted an investigation, which was unable to confirm the accuracy of the authorship list and the origins of the study. Furthermore, an inappropriately high level of overlap with existing literature, without appropriate citation, was identified.

Consequently, the Editorial Board has lost confidence in the integrity of the findings and has decided to retract this publication [1] as per MDPI’s retraction policy (https://www.mdpi.com/ethics#_bookmark30).

This retraction was approved by the Editor-in-Chief of the *Journal of Cardiovascular Development and Disease*.

Javid Ahmad Malik wishes to indicate that he was not aware of this submission nor consented to its publication.

The authors did not provide a comment on this decision.
